# No benefit of longer eradication therapy of *Pseudomonas aeruginosa* primoinfections in pediatric cystic fibrosis

**DOI:** 10.1186/s13104-019-4157-8

**Published:** 2019-03-04

**Authors:** F. Claude, I. Rochat, G. M. Hafen

**Affiliations:** 1 Faculty of Biology and Medicine, University of Lausanne, Lausanne, Switzerland; 2 Department of Pediatrics, Respiratory Unit, Centre Hospitalier Universitaire Vaudois, Lausanne, Switzerland

**Keywords:** Cystic fibrosis, *Pseudomonas aeruginosa*, Primoinfection, Eradication protocol

## Abstract

**Objective:**

Patients with cystic fibrosis are more susceptible than members of the general population to lung infections. Infections with *Pseudomonas aeruginosa* require particular attention, because they may accelerate the deterioration of lung function if not adequately treated. This study assessed the eradication rate of *P. aeruginosa* primoinfections, with a protocol of inhaled tobramycin and oral ciprofloxacin over a 3 months’ period.

**Results:**

Retrospective single-center study from June 1st, 2007 to December 31st, 2015. Inclusion of 28 pediatric patients (11 females, 17 males), with a total of 49 primoinfections. Overall success rate of 67.3%, which is similar or even inferior to figures published in the literature.

## Introduction

Airways infections in patients with cystic fibrosis (CF) may deteriorate their lung function, particularly those involving *Pseudomonas aeruginosa* [1]. The early detection of this pathogen is fundamental, because its phenotypic changes and the exacerbated inflammatory response across the time render its eradication more difficult [1–5].

At the pediatric CF outpatient clinic at Lausanne University Hospital, the first detection ever or the reacquisition of *P. aeruginosa* after a minimum of 6 months and at least three negative bacterial cultures was defined as “primoinfection” and was treated with inhaled tobramycin and oral ciprofloxacin over 3 months. To our knowledge, this is one of the longest therapeutic interventions described [6–11]. With this study, we wanted to determine if a longer protocol brings benefits in terms of eradication.

## Main text

### Method

#### Intervention

**Fig. 1:**
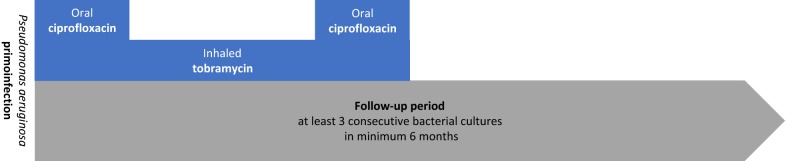
Eradication protocol and follow-up

We treated *P. aeruginosa* primoinfections with three consecutive cycles of 28 days of inhaled tobramycin (TOBI^®^ by Novartis or Bramitob^®^ by Chiesi; 300 mg BID), with oral ciprofloxacin (Ciproxin^®^ by Bayer; 30 to 40 mg/kg/day in two doses, maximum 1500 mg/day) during the first and last 21 days of the protocol (Fig. 1). We evaluated the efficacy after a 6 months follow-up (3 months of therapy, 3 months post-therapy) by a multidisciplinary team (physicians, physiotherapists, dieticians, CF nurse, social worker and psychiatrist), with collection of minimum three bacterial cultures (the latest always at the end of the follow-up). A success in eradication was defined by negative cultures for *P. aeruginosa* during the whole follow-up. A failure in eradication was defined by at least one positive culture for *P. aeruginosa*. In this case or by deterioration of clinical conditions of our patients, other therapeutic options were evaluated (for instance, intra-venous antibiotic treatment).

#### Outcomes

Determination of the eradication rate of our eradication protocol. Calculation of mean ages, assessment of symptoms and methods used to obtain the cultures at the primoinfection. Analyze of the evolution of the lung function and the anthropometric parameters, comparing the best values in the 6 months before the primoinfection and during the 6 months after the initiation of the eradication protocol. Assessment of most common bacterial coinfections at the primoinfection and at the end of the follow-up.

#### Design

Retrospective study in the pediatric CF outpatient clinic at Lausanne University Hospital. In a preliminary study in 2011, we analyzed data from 20 primoinfections treated with our eradication protocol since June 1st, 2007, eradication rate of 80%. To confirm this result, according to the size of our outpatient clinic (about 60 patients), we estimated a minimum needed of 49 primoinfections (confidence interval 95%, margin of error 5%) to gather till December 31st, 2015. Pediatric outpatients (up to 18 years old), with a confirmed diagnosis of CF (by a positive sweat test and/or two disease-causing mutations) and with *P. aeruginosa* primoinfections were included. Patients with *P. aeruginosa* primoinfections not exactly treated according to the protocol, with an initial intra-venous antibiotic course followed by the protocol or with incomplete data sets were excluded.

#### Data

Sex, common symptoms and age at the primoinfection diagnosis, diagnostic methods used to obtain the cultures, forced expiratory volume in 1 s (FEV1), body mass index (BMI) and data about the most common bacterial coinfections were collected in a FileMaker Pro 15 database. Statistical analysis with Xlstat-Biomed 19.5 and Microsoft Excel 2016. According to the data distribution (normal or not), we used parametric (T test) or non-parametric (Mann–Whitney, Wilcoxon or McNemar) tests.

### Primary outcome

Our study included 28 patients (11 females, 17 males), with total of 49 primoinfections. 33 primoinfections successfully treated, with an overall success rate of 67.3% (confidence interval 54.2–80.4%). Success rate of 78.9% for the girls and 60.0% for the boys, statistically different (p = 0.006, McNemar). However, mean number of primoinfections between the girls (1.727) and the boys (1.765) not statistically different (p = 0.789, Mann–Whitney).

In the success group, 2 patients had 2 primoinfections, 2 patients had 3 primoinfections. Moreover, 3 patients had 2 primoinfections successfully treated and 1 failure in eradication. In the failure group, 1 patient had 4 primoinfections.

### Secondary outcomes

#### Mean ages

9.3 years in the success group (SD 4.5 years) and 9.1 years in the failure group (SD 3.7 years), no statistical difference (p = 0.842, T test) (Table 1). According to the sex, 10.1 years (SD 4.6 years) for the girls and 8.3 years for the boys (SD 4.1 years), no statistical difference (p = 0.290, T test).

**Table 1:** Secondary outcomes

	Success group		Failure group	
Primoinfections [n]	33		16	
Age at diagnosis [years]	9.3		9.1	
	p value 0.842 (T test)			
Symptoms at diagnosis [%]	81.8		87.5	
	p value 0.118 (McNemar)			
Cough [%]	*66.7*		*87.5*	
Upper respiratory tract infect. [%]	*39.4*		*25.0*	
Dyspnea [%]	*3.0*		*12.5*	
Fever [%]	*3.0*		*12.5*	
Diagnostic methods [%]				
Sputum samples	69.7		75.0	
Oropharyngeal swab	30.3		25.0	
	p value 0.831 (McNemar)			

	Diagn.	Follow-up	Diagn.	Follow-up
FEV1 [%]				
Mean	107.7	107.8	104.7	99.1
Median	103	103	104	99.5
p value	0.772 (Wilcoxon)		0.312 (T test)	
BMI [kg/m^2^]				
Mean	16.3	16.6	16.9	17.1
Median	15.8	16.0	16.6	16.2
p value	0.024 (Wilcoxon)		0.083 (Wilcoxon)	

Among symptomatic patients, distribution of most frequent symptoms. A patient may have more than one symptom (in italics)

#### Symptoms

27 patients in the success group (81.8%) and 14 in the failure group (87.5%) were symptomatic at the primoinfection diagnosis, no statistical difference between the groups (p = 0.118, McNemar). Some patients had more than one symptom (Table 1).

#### Diagnostic methods

Material for *P. aeruginosa* cultures was gathered in the majority of the cases through sputum samples, then through oropharyngeal swabs. No other methods were used (for instance, bronchoalveolar lavage). No significant difference between these two methods (Table 1).

#### Lung function evolution

We didn’t observe any significant changes in the mean FEV1 value before and after the eradication protocol (Table 1).

#### Anthropometric evolution

We observed an increase of the mean BMI value before and after the eradication protocol for the two groups, not statistically significant in the failure group (Table 1).

#### Bacterial coinfections

**Fig. 2:**
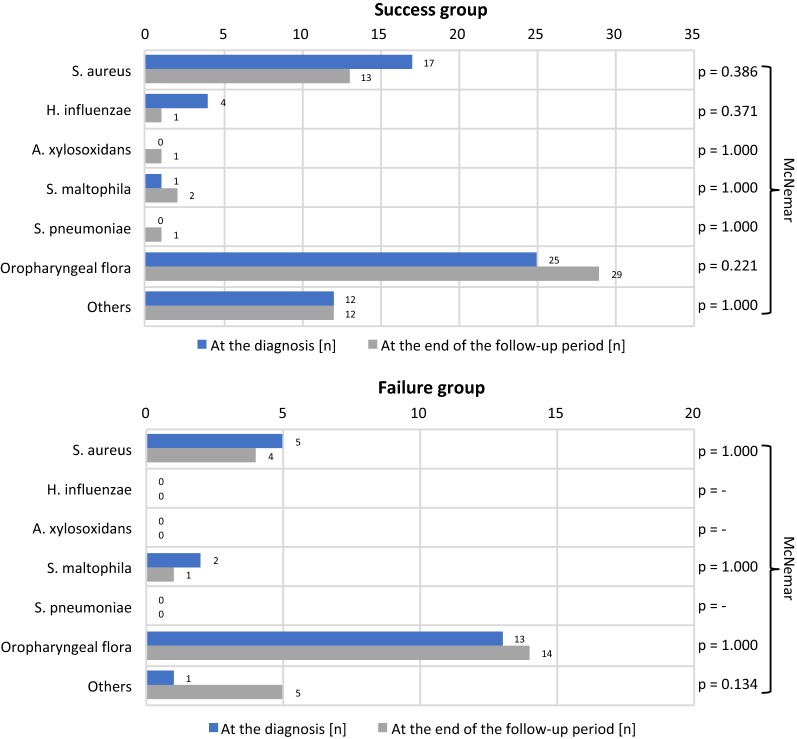
Bacterial co-infections

The physiological oropharyngeal flora was evidenced in most of the primoinfections through the study. The co-pathogen observed most frequently at the primoinfection was *Staphylococcus aureus* (methicillin sensitive), followed by *Haemophilus influenzae* and *Stenotrophomonas maltophilia*. At the end of the follow-up, we noticed a non-statistically significative diminution of these pathogens and a non-statistically significative emergence of *Achromobacter xylosoxidans* and *Streptococcus pneumoniae* in the success group. Some other co-pathogens were rare and classified as “other”. Their incidence didn’t vary through the study (Fig. 2).

### Discussion

Our retrospective study demonstrated that a 3 months’ therapy in case of *P. aeruginosa* primoinfections brings no extra benefits in terms of eradication. Our overall success rate was 67.3%. We noticed a significant difference between the girls (78.9%) and the boys (60.0%), attributed to a better compliance of the girls.

The definition of successful eradication, the duration of inhaled therapy, the addition of oral ciprofloxacin and the nature of follow-up remain matter of debate [12]. In the United States, the CF Foundation recommends 28 days of inhaled tobramycin [13]. About the duration of the inhaled therapy, Ratjen et al. assessed the time to recurrence of *P. aeruginosa* after 28 or 56 days of inhaled tobramycin. It was similar in the two groups (26.12 months with 28 days and 25.82 months with 56 days). At the end of the study, 66% of patients were free from *P. aeruginosa* with 28 days and 69% with 56 days [7]. Gibson et al. assessed the *P. aeruginosa* eradication from the lower airways after 28 or 56 days of inhaled tobramycin, with different times of follow-up. It was similar in all groups—globally 75% [6]. Most recently, Blanchard et al. analyzed a three-steps eradication strategy. In asymptomatic patients with new onset of *P. aeruginosa*, the eradication rate was 76.9% after a first cycle of 28 days of inhaled tobramycin. Successive steps could improve the eradication rate [11]. The use of oral ciprofloxacin was assessed by Treggiari et al. They compared four different regimens that combined cyclic or culture-based inhaled tobramycin, with oral ciprofloxacin or a placebo for 14 days. 17 patients of 133 were *P. aeruginosa* positive at the end of the study in the ciproxins’ group and 12 of 134 in the placebos’ group [8]. The use of inhaled tobramycin vs colistin was addressed by Taccetti et al. and Proesmann et al. [9, 10]. The first study analyzed the efficacy of 28 days of inhaled tobramycin or colistin, with oral ciprofloxacin. Success in eradication was defined by successive negative cultures in 6 months and was similar among the groups (colistin 62.8%, tobramycin 65.2%) [9]. The second study compared 3 months of inhaled colistin and oral ciprofloxacin to 28 days of inhaled tobramycin. At the end of the treatment, the eradication rates were similar among the groups (colistin and ciprofloxacin 89.7%, tobramycin 79.3%) [10]. For the studies that assessed the eradication rates [6, 9–11], they were similar or even better than ours (67.3%). Most of these pediatric studies used shorter inhaled therapies [6–9, 11]. One of them demonstrated that the use of oral ciprofloxacin brings no extra benefits [8]. Others showed that the use of inhaled tobramycin or colistin is equivalent [9, 10]. These findings are fundamental, because reducing the duration of the inhaled therapy, avoiding to give oral ciprofloxacin, having the choice between tobramycin and colistin, could increase the compliance of patients, decrease the risk of side-effects and last but not least the costs of the therapy. As a consequence, we decided to reduce our eradication protocol to 1 month of inhaled tobramycin or colistin. However, according to the guidelines of the Swiss Working Group of Cystic Fibrosis, our patients are still treated in association with oral ciprofloxacin [14].

Our analysis of secondary outcomes revealed no significant difference between groups in terms of age, as reported in a previous study (studies) [7]. Most of our patients were symptomatic at the time of the diagnosis. In a (some) previous study (studies), patients with acute lung disease were excluded [7]. The material used to derive *P. aeruginosa* cultures was gathered in the majority in the form of sputum samples. Bronchoalveolar lavage as a “gold standard” for identification of lower respiratory tract infections in patients with cystic fibrosis was recently discussed [15–18]. The results obtained with bronchoalveolar lavage are similar to those obtained with induced sputum [19]. In addition to the technical issues of bronchoalveolar lavage (general anesthesia in children), samples obtained vary greatly, depending on localization within the lungs [20–22]. As reported previously, evaluation of the lung function (FEV1) before implementation of the eradication protocol were similar to those obtained after its completion [7]. We noticed an increase in BMI over the course of the study, but not statistically significant because of the failure group. Regarding the co-pathogens, one study showed that bacterial co-infections at the time of primoinfection were not a risk factor for failed eradication [11]. The co-pathogens observed most frequently at the primoinfection diagnosis were *S. aureus* and *H. influenzae*. This observation is similar to findings reported previously [6–9]. In our study, *S. maltophilia* was the third most frequent pathogen identified at the primoinfection diagnosis. However, previous studies reported its identification during the follow-up [8, 9]. We observed the emergence of *A. xylosoxidans* and *S. pneumoniae* in some cases, but this trend was not statistically significant.

### Conclusion

The overall eradication rate of *P. aeruginosa* primoinfection with use of our 3 months’ protocol was 67.3%. This figure is similar or even inferior to those reported previously, despite our study’s use of a longer duration of inhaled therapy with tobramycin as well as combination treatment with oral ciprofloxacin. These findings elucidate the nature of patient compliance, side effects and economic considerations.

## Limitations

- Small sample size and retrospective design of our study, that prevented a stratification of our patients and risk to draw general conclusions for bigger cohorts followed in CF centers across the world. However, our conclusions are supported by other studies which use shorter protocols [6–9, 11].
- Comparison across studies is difficult because of methodological discrepancies and lack of standardized definitions.
- During the 6 months’ follow-up, patients are grouping according to the result of their *P. aeruginosa* cultures. Keeping outpatients in a positive cohort for such a long time can lead to cross-infections.
- No genotyping of *P. aeruginosa*, in order to separate precisely a new primoinfection and the persistence of the pathogen.

## Abbreviation

CF: cystic fibrosis

## References

[1] Stuart B, Lin JH, Mogayzel PJ (2010). Early eradication of *Pseudomonas aeruginosa* in patients with cystic fibrosis. Paediatr Respir Rev.

[2] Bush A (2002). Decisions facing the cystic fibrosis clinician at first isolation of *Pseudomonas aeruginosa*. Paediatr Respir Rev.

[3] Bassinet L (2003). Strategy of antibiotic therapy in the course of chronic *Pseudomonas aeruginosa* infection. Rev Mal Respir.

[4] Koch C, Cuppens H, Rainisio M, Madessani U, Harms H, Hodson M, Mastella G, Navarro J, Strandvik B, McKenzie S (2001). European Epidemiologic Registry of Cystic Fibrosis (ERCF): comparison of major disease manifestations between patients with different classes of mutations. Pediatr Pulmonol.

[5] Eber E, Zach MS (2010). *Pseudomonas aeruginosa* infection in cystic fibrosis: prevent, eradicate or both?. Thorax.

[6] Gibson RL, Emerson J, Mayer-Hamblett N, Burns JL, McNamara S, Accurso FJ, Konstan MW, Chatfield BA, Retsch-Bogart G, Waltz DA (2007). Duration of treatment effect after tobramycin solution for inhalation in young children with cystic fibrosis. Pediatr Pulmonol.

[7] Ratjen F, Munck A, Kho P, Angyalosi G, ELITE Study Group (2010). Treatment of early *Pseudomonas aeruginosa* infection in patients with cystic fibrosis: the ELITE trial. Thorax.

[8] Treggiari MM, Retsch-Bogart G, Mayer-Hamblett N, Khan U, Kulich M, Kronmal R, Williams J, Hiatt P, Gibson RL, Spencer T (2011). Comparative efficacy and safety of 4 randomized regimens to treat early *Pseudomonas aeruginosa* infection in children with cystic fibrosis. Arch Pediatr Adolesc Med.

[9] Taccetti G, Bianchini E, Cariani L, Buzzetti R, Costantini D, Trevisan F, Zavataro L, Campana S, Italian Group for P aeruginosa Eradication in Cystic Fibrosis (2012). Early antibiotic treatment for *Pseudomonas aeruginosa* eradication in patients with cystic fibrosis: a randomised multicentre study comparing two different protocols. Thorax.

[10] Proesmans M, Vermeulen F, Boulanger L, Verhaegen J, De Boeck K (2013). Comparison of two treatment regimens for eradication of *Pseudomonas aeruginosa* infection in children with cystic fibrosis. J Cyst Fibros.

[11] Blanchard AC, Horton E, Stanojevic S, Taylor L, Waters V, Ratjen F (2017). Effectiveness of a stepwise *Pseudomonas aeruginosa* eradication protocol in children with cystic fibrosis. J Cyst Fibros.

[12] Langton Hewer SC, Smyth AR. Antibiotic strategies for eradicating *Pseudomonas aeruginosa* in people with cystic fibrosis. In: Cochrane database of systematic reviews. John Wiley & Sons, Ltd; 2017. http://onlinelibrary.wiley.com/doi/10.1002/14651858.CD004197.pub5/abstract. 10.1002/14651858.cd004197.pub5. Accessed 1 Nov 2017.

[13] Mogayzel PJ, Naureckas ET, Robinson KA, Brady C, Guill M, Lahiri T, Lubsch L, Matsui J, Oermann CM, Ratjen F (2014). Cystic Fibrosis Foundation pulmonary guideline. Pharmacologic approaches to prevention and eradication of initial *Pseudomonas aeruginosa* infection. Ann Am Thorac Soc.

[14] Regamey N. Treatment of *Pseudomonas aeruginosa* (PA) infection in CF patients, Swiss Working Group Cystic Fibrosis (SWGCF) protocol—30.06.2009. 2018.

[15] Ramsey BW, Wentz KR, Smith AL, Richardson M, Williams-Warren J, Hedges DL, Gibson R, Redding GJ, Lent K, Harris K (1991). Predictive value of oropharyngeal cultures for identifying lower airway bacteria in cystic fibrosis patients. Am Rev Respir Dis.

[16] Armstrong DS, Grimwood K, Carlin JB, Carzino R, Olinsky A, Phelan PD (1996). Bronchoalveolar lavage or oropharyngeal cultures to identify lower respiratory pathogens in infants with cystic fibrosis. Pediatr Pulmonol.

[17] Rosenfeld M, Emerson J, Accurso F, Armstrong D, Castile R, Grimwood K, Hiatt P, McCoy K, McNamara S, Ramsey B (1999). Diagnostic accuracy of oropharyngeal cultures in infants and young children with cystic fibrosis. Pediatr Pulmonol.

[18] Aaron SD, Kottachchi D, Ferris WJ, Vandemheen KL, Denis MLS, Plouffe A, Doucette SP, Saginur R, Chan FT, Ramotar K (2004). Sputum versus bronchoscopy for diagnosis of *Pseudomonas aeruginosa* biofilms in cystic fibrosis. Eur Respir J.

[19] Blau H, Linnane B, Carzino R, Tannenbaum E-L, Skoric B, Robinson PJ, Robertson C, Ranganathan SC (2014). Induced sputum compared to bronchoalveolar lavage in young, non-expectorating cystic fibrosis children. J Cyst Fibros.

[20] Gutierrez JP, Grimwood K, Armstrong DS, Carlin JB, Carzino R, Olinsky A, Robertson CF, Phelan PD (2001). Interlobar differences in bronchoalveolar lavage fluid from children with cystic fibrosis. Eur Respir J.

[21] Willner D, Haynes MR, Furlan M, Schmieder R, Lim YW, Rainey PB, Rohwer F, Conrad D (2012). Spatial distribution of microbial communities in the cystic fibrosis lung. ISME J.

[22] Gilchrist FJ, Salamat S, Clayton S, Peach J, Alexander J, Lenney W (2011). Bronchoalveolar lavage in children with cystic fibrosis: how many lobes should be sampled?. Arch Dis Child.

